# Supplementary material for: Determination of the Amino Acid Recruitment Order in Early Life by Genome-Wide Analysis of Amino Acid Usage Bias
Source: Biomolecules. 2022 Jan 21;12(2):171. doi: 10.3390/biom12020171 (PMC8961565; doi:10.3390/biom12020171)
Supplement: Supplementary file 1 [file biomolecules-12-00171-s001.zip › Supplementary Figures 1-4.pdf]

Supplementary Figures:

Determination of the Amino Acid Recruitment Order in Early Life by Genome-wide Analysis of Amino Acid Usage Bias

Ming-Xiao Zhao[1], Ruo-Fan Ding[2], Yan Liu[1, *], Zhi-Liang Ji[2, *], Yu-Fen Zhao[1, 3, *]

[1] Department of Chemical Biology, Key Laboratory for Chemical Biology of Fujian Province, College of Chemistry and Chemical Engineering, Xiamen University, Xiamen, Fujian, 361005, P. R. China

[2] State Key Laboratory of Cellular Stress Biology, School of Life Science, Xiamen University, Xiamen 361102, P. R. China

[3] Institute of Drug Discovery Technology, Ningbo University, Ningbo, Zhejiang, 315221, P. R. China

Supplementary file contains:

Supplementary Figure 1

Supplementary Figure 2

Supplementary Figure 3

Supplementary Figure 4

Supplementary Figure 1

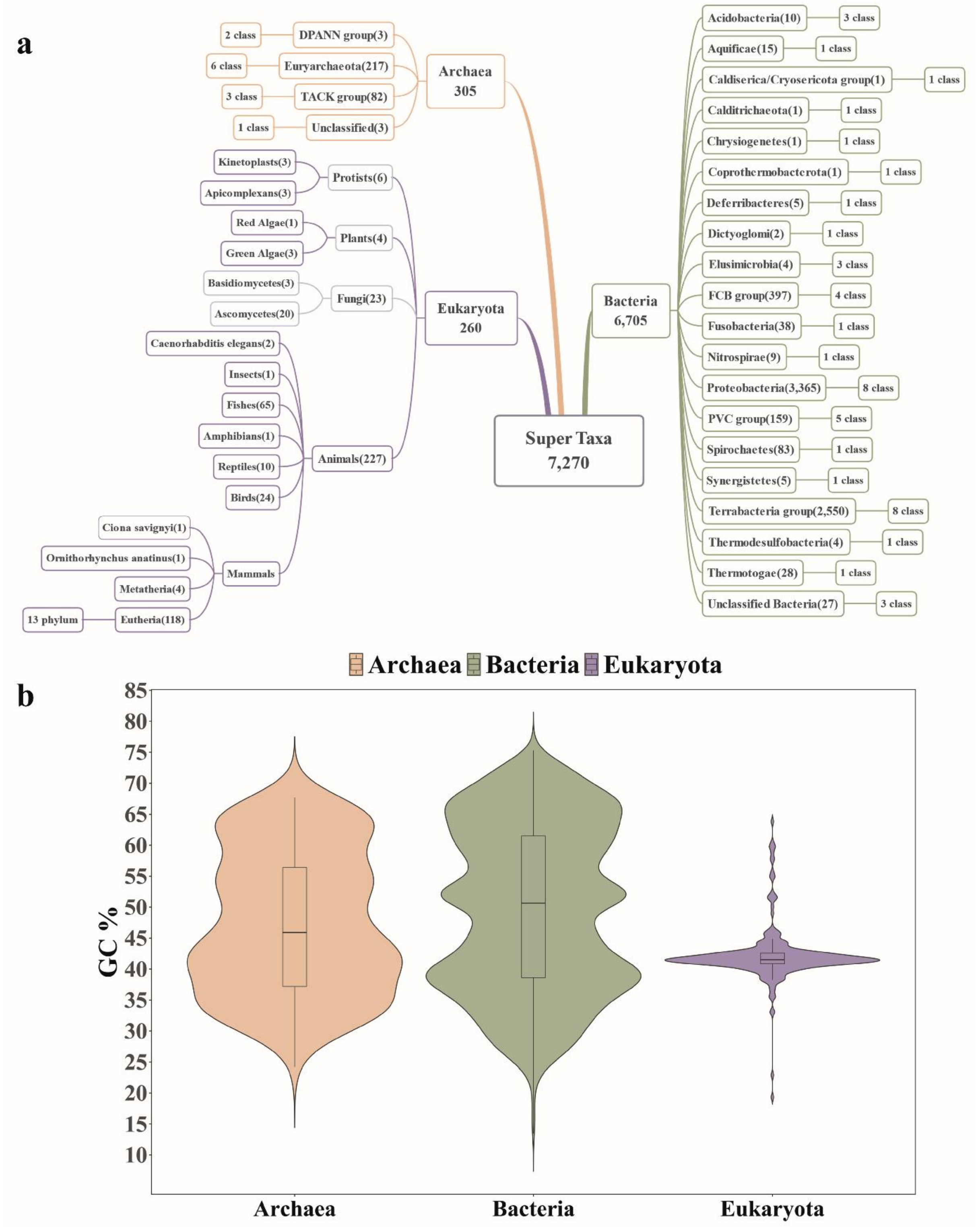

Figure S1. Information on the species in this study. (a) Spezies classification. The number indicates the number of species included in this category. **(b)** Genomic GC content distribution in three domains of life.

Supplementary Figure 2

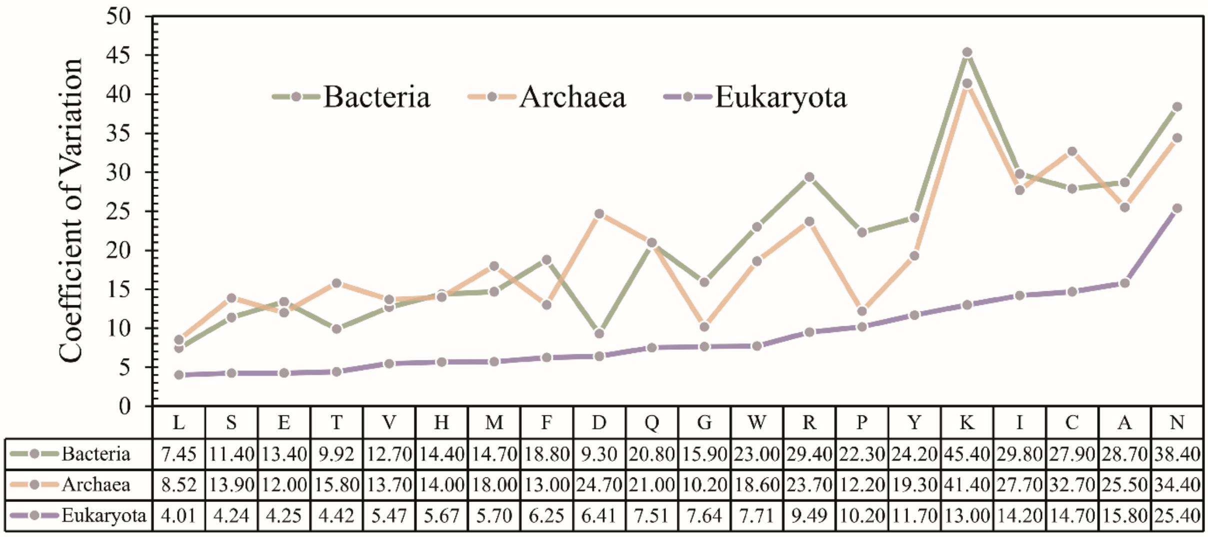

	L	S	E	T	V	H	M	F	D	Q	G	W	R	P	Y	K	I	C	A	N
Bacteria	7.45	11.40	13.40	9.92	12.70	14.40	14.70	18.80	9.30	20.80	15.90	23.00	29.40	22.30	24.20	45.40	29.80	27.90	28.70	38.40
Archaea	8.52	13.90	12.00	15.80	13.70	14.00	18.00	13.00	24.70	21.00	10.20	18.60	23.70	12.20	19.30	41.40	27.70	32.70	25.50	34.40
Eukaryota	4.01	4.24	4.25	4.42	5.47	5.67	5.70	6.25	6.41	7.51	7.64	7.71	9.49	10.20	11.70	13.00	14.20	14.70	15.80	25.40

Figure S2. Coefficient of variation of amino acid usage in three domains of life. The coefficient of variation (CV) is a normalized measure of the dispersion degree of the probability distribution, which is defined as the ratio of the standard deviation (SD) to the average value.

Supplementary Figure 3

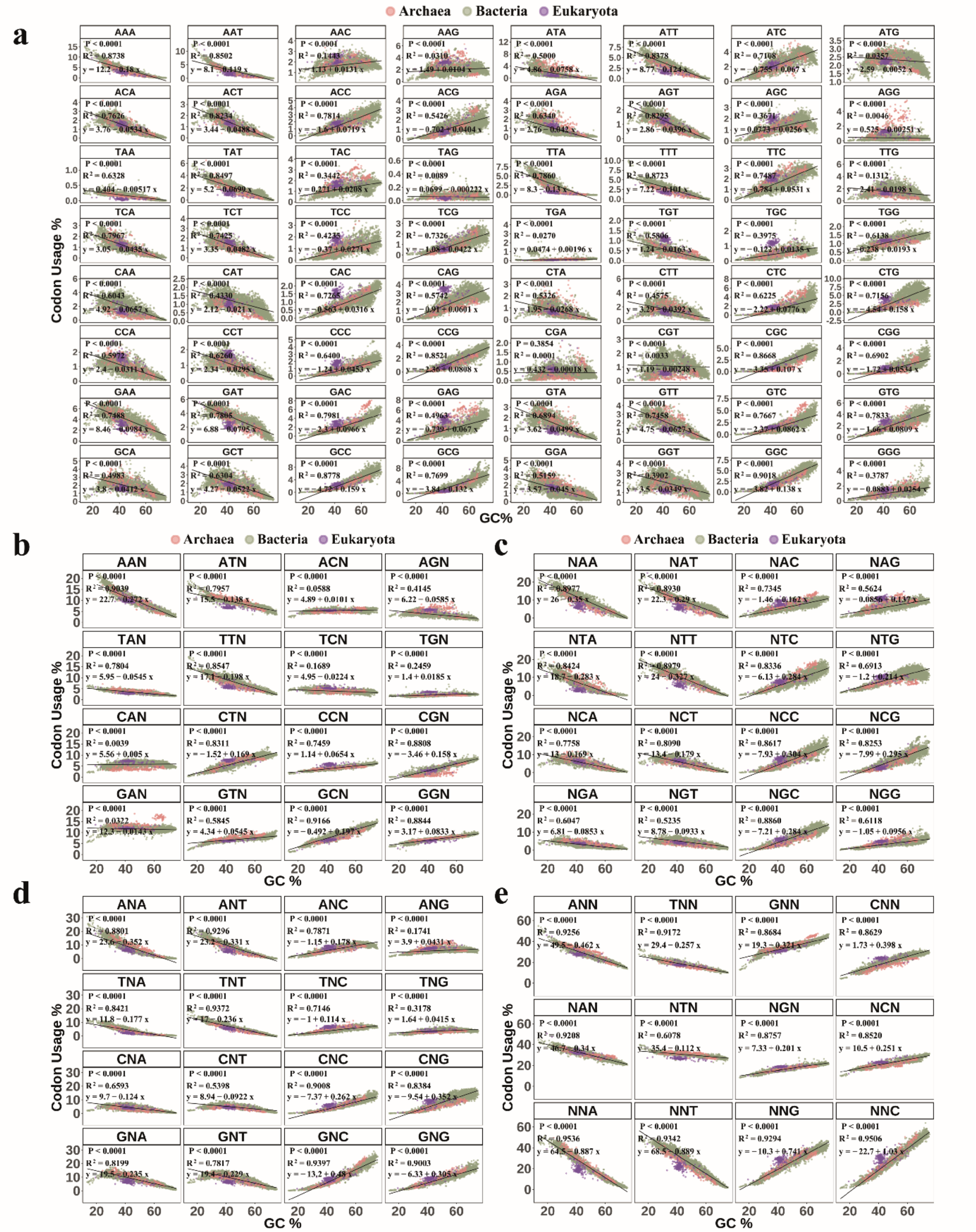

Figure S3. Correlation between the species' GC content and codon usage. **(a)** 64 codons; **(b)** codons with the same first two bases; **(c)** codons with the same last two bases; **(d)** codons with the same first and third bases; **(e)** codons with the same one base. The abscissa represents GC content, and the ordinate represents the codon usage (%).

Supplementary Figure 4

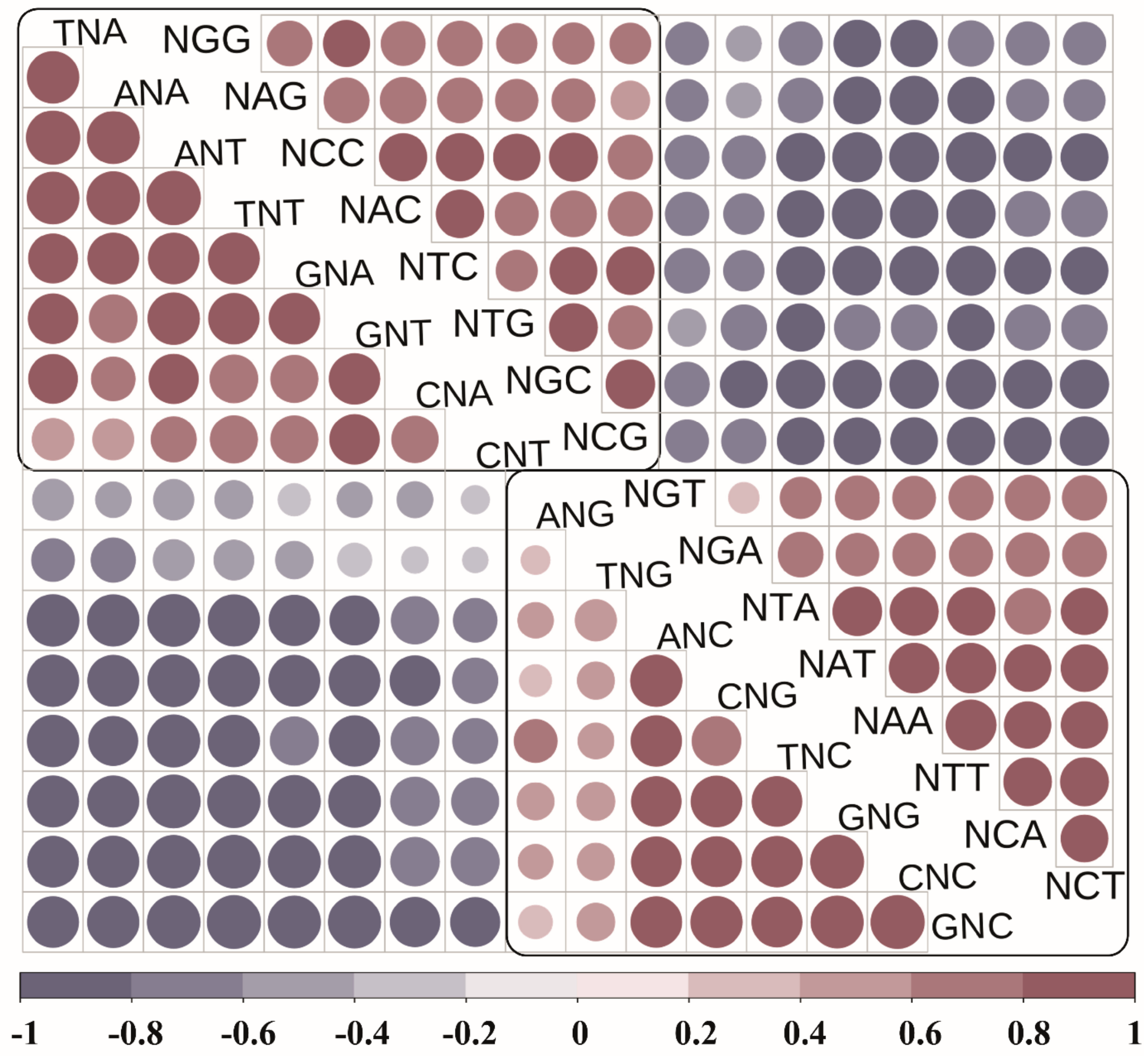

Figure S4. Correlation of codon usage with the same last two bases (lower) and the same first and third bases (upper).
